# Conocimientos de soporte vital básico y primeros auxilios en el alumnado de educación primaria y educación secundaria obligatoria

**DOI:** 10.15446/rsap.V27n5.115766

**Published:** 2025-09-01

**Authors:** Brais Ruibal-Lista, Alicia Fernández-Sanabria, Carla Carta-Monje, Sergio López-García

**Affiliations:** 1 BR: Ciencias del Deporte y la Educación Física. Ph. D. Ciencias del Deporte y la Educación Física. EUM Fray Luis de León. Universidad Católica de Ávila. Ávila, España. brais.ruibal@frayluis.com Universidad Católica de Ávila Universidad Católica de Ávila Ávila Spain; 2 AF: Educación Primaria. Docente, Educación Infantil y Educación Primaria. EUM Fray Luis de León. Universidad Católica de Ávila. Ávila, España. alicia.fernández.sanabria@frayluis.com Universidad Católica de Ávila Universidad Católica de Ávila Ávila Spain; 3 CC: Educación Primaria. Docente, Educación Infantil y Educación Primaria. EUM Fray Luis de León. Universidad Católica de Ávila. Ávila, España. carla.carta.monje@frayluis.com Universidad Católica de Ávila Universidad Católica de Ávila Ávila Spain; 4 SL: Ciencias de la Actividad Física y el Deporte. Catedrático de Universidad, Facultad de Educación, Universidad Pontificia de Salamanca. Salamanca, España. slopezga@upsa.es Universidad Pontificia de Salamanca Facultad de Educación Universidad Pontificia de Salamanca Salamanca Spain slopezga@upsa.es

**Keywords:** Primeros auxilios, accidente, escuela, formación, profesorado, RCP *(fuente: DeCS, BIREME)*, First aid, accident, school, training, teaching staff, CPR *(source: MeSH, NLM)*

## Abstract

**Objetivo:**

La formación desde temprana edad en reanimación cardiopulmonar (RCP) y primeros auxilios es crucial debido a la alta incidencia de muerte súbita cardiaca. Este estudio analiza los conocimientos en soporte vital básico y primeros auxilios entre estudiantes de educación primaria y secundaria obligatoria en Valladolid.

**Métodos:**

Participaron 240 estudiantes de 10 a 14 años mediante un cuestionario que abarcó dimensiones sociodemográficas, conocimientos generales de SVB, específicos de RCP en adultos y uso del desfibrilador externo automático (DEA).

**Resultados:**

Los resultados indican que los alumnos de primaria tienen conocimientos limitados sobre SVB, con solo el 8,6% capaz de explicar correctamente el masaje cardíaco y el 22,9% la profundidad adecuada de compresiones. En secundaria, aunque el conocimiento es ligeramente mejor, sigue siendo insuficiente. Por otro lado, la formación previa mostró una influencia significativa en los conocimientos demostrados.

**Conclusión:**

Los estudiantes de educación primaria y educación secundaria obligatoria presentan bajos niveles de conocimiento en SVB, subrayando así la necesidad de mejorar la formación en estas habilidades.

## Abstract (en)

**Objective:**

Early training in cardiopulmonary resuscitation (CPR) and first aid is crucial due to the high incidence of sudden cardiac death. This study examines the knowledge of basic life support and first aid among Primary and Secondary Education students in Valladolid.

**Methods:**

A total of 240 students aged 10 to 14 participated through a questionnaire covering sociodemographic dimensions, general BLS knowledge, specific knowledge of adult CPR, and the use of an automated external defibrillator (AED).

**Results:**

The results indicate that Primary school students have limited knowledge of BLS, with only 8.6% able to correctly explain cardiac massage and 22.9% understanding the appropriate compression depth. In Secondary school, although knowledge is slightly better, it remains insufficient. Furthermore, prior training showed a significant influence on demonstrated knowledge.

**Conclusions:**

Primary and Secondary Education students exhibit low levels of BLS knowledge, highlighting the need to improve training in these skills.

La muerte súbita cardiaca es una de las principales causas de fallecimiento en los países industrializados, lo que subraya la importancia de la formación en RCP. Se ha estimado que la optimización y la difusión generalizada de estas habilidades pueden ser cruciales. A los ciudadanos se les debe instruir en la RCP básica, ya que, según el European Resuscitation Council 1, la rapidez con la que se inician las maniobras de reanimación en el lugar del incidente y su correcta ejecución, son factores cruciales para prevenir secuelas posteriores e incluso la muerte de la víctima.

Se ha comprobado que las probabilidades de supervivencia y de mantener una buena calidad de vida después de un paro cardiaco dependen del tiempo que se tarde en iniciar las maniobras de reanimación 2. Una intervención adecuada en los primeros 3-5 minutos puede aumentar las tasas de supervivencia 3. Generalmente, estas acciones son llevadas a cabo por personas capacitadas en RCP, que no necesariamente pertenecen al ámbito médico 4.

Por todo esto, desde hace años se considera el centro escolar como el lugar idóneo para iniciar a toda la población en materia de primeros auxilios y RCP. Se ha podido observar que la formación en PPAA podría dar comienzo ya en la etapa infantil, en niños de segundo ciclo de educación infantil (3-6 años), ya que tienen leves conocimientos del número de emergencias, la valoración de la conciencia y la respiración, la posición lateral de seguridad o la transmisión de información a servicios de emergencias 5.

A partir de la edad de siete años, los niños son capaces de aprender, reproducir y retener los conocimientos teóricos en relación con la maniobra de RCP 6; a los nueve años son capaces de poner en práctica 7; a los diez son capaces de realizar compresiones torácicas adecuadas en un maniquí 8 y, a los trece años pueden lograr una calidad de RCP similar a la de una persona adulta 9.

Además de la parada cardíaca, se ha observado que existe un gran número de accidentes dentro del ámbito escolar, los cuales están ligados a la práctica y la actividad física y, por lo tanto, a la asignatura de Educación Física 10,11.

Los accidentes más comunes que pueden producirse en el ámbito educativo son: caídas, choques y colisiones, cortes, daños musculares, quemaduras, pinchazos, heridas, hemorragias, atragantamientos o asfixias, intoxicaciones y electrocuciones. Estos accidentes aumentan con la edad de los escolares; son más frecuentes en chicos que en chicas; el lugar con mayor riesgo de accidentes es el patio; y las actividades que mayor riesgo conllevan son las que se desarrollan en el recreo o durante las sesiones de Educación Física 12,13.

Es necesario indicar que el aprendizaje de los primeros auxilios está justificado, además, por la evidencia científica, por su presencia obligatoria dentro del currículo educativo en la asignatura Educación Física, tanto en educación primaria 14 como en educación secundaria obligatoria 15, donde destacan contenidos como la conducta PAS, el protocolo de SVB, las maniobras de RCP o el uso del DEA 16.

Por todo esto, el objetivo de este estudio fue analizar los conocimientos de soporte vital básico (SVB) y primeros auxilios en el alumnado de educación primaria y ESO de Valladolid.

## MÉTODO

Fueron invitados a participar dos centros educativos concertados de Valladolid, España. La población objeto de estudio fue el alumnado de último ciclo de educación primaria y primer ciclo de educación secundaria obligatoria (entre 10 y 14 años).

### Procedimiento

En primer lugar, se tomó contacto con el equipo directivo de cada uno de los centros educativos participantes, explicando los objetivos del estudio y solicitando su colaboración. Una vez aceptada la participación de los centros, se le explicó al profesorado de Educación Física el procedimiento del estudio.

A continuación, se solicitó el consentimiento informado a los padres o tutores legales de los menores para permitir su participación. Una vez se recibieron los consentimientos, se concertó un día con cada centro educativo y grupo para recoger los datos de esta investigación de manera presencial.

### Herramientas y medidas

Para evaluar los conocimientos en SVB se utilizó un cuestionario empleado en otros estudios con profesorado en ejercicio de estas mismas etapas educativas 17-18, y maestros en formación 19. Las preguntas que se formularon en el cuestionario fueron redactadas con base en cuatro dimensiones:

- Sociodemográfica: cuatro abiertas (iniciales de nombre y apellidos; fecha de nacimiento; género y curso), dos dicotómicas ([sí/no]: formación previa sobre SVB y tiempo transcurrido desde la formación: hace menos de un año-hace más de un año) y una escala Likert ([de 0-4, de ningunos (0) a excelentes (4)]; consideración del nivel de conocimientos).
- Conocimientos generales en SVB: una cerrada ([correcto-incorrecto]: secuencia de actuación ante persona en posible parada cardiaca: 1. Seguridad de la escena; 2. Comprobación de la conciencia; 3. Comprobación de la respiración; 4. Llamada al servicio de emergencias; 5. Comienzo de compresiones cardiacas externas; 6. Aplicación del DEA); cuatro abiertas ([correcto-incorrecto]: verificación de conciencia/inconsciencia; apertura de la vía aérea (maniobra); evaluación de la respiración (forma y tiempo); teléfono de emergencias.
- Conocimientos específicos de RCP en adultos: una dicotómica ([sí-no]: conocimiento de masaje cardiaco externo en adultos) y cuatro abiertas ([correcto-incorrecto]: lugar de colocación de manos; fracción de compresiones torácicas/ventilación; frecuencia de compresiones (compresiones/minuto); profundidad de las compresiones torácicas.
- Conocimiento y uso del DEA: tres dicotómicas ([sí-no]: conocimiento del DEA; conocimiento del uso; intención de uso en caso necesario) y una cerrada ([correcto/incorrecto]: orden correcto de ejecución del DEA: 1. Encender, 2. Colocar parches, 3. Insertar el conector de parches, 4. Seguir instrucciones, 5. Descarga). Para medir el nivel de conocimientos global de los escolares, se creó un sistema de puntuación que otorgó un punto por cada respuesta correcta (nueve preguntas de RCP y una del DEA).

### Aspectos éticos

La investigación fue aprobada por la Comisión de Calidad de la Escuela Universitaria de Magisterio Fray Luis de León, adscrita a la Universidad Católica de Ávila. Toda la investigación se llevó a cabo de acuerdo con la Declaración de Helsinki.

### Análisis estadístico

Los datos de los cuestionarios se introdujeron en el software estadístico SPSS (SPSS v.26, IBM Corporation, Nueva York, EE. UU.) para su posterior tratamiento. Los resultados de cada variable se expresan en frecuencias absolutas y relativas (porcentajes).

Para analizar las diferencias entre las variables demográficas y dicotómicas se empleó el estadístico T student para muestras independientes o el estadístico Mann-Whitney, en función de la normalidad de los datos. Posteriormente, se realizó un análisis estadístico utilizando tablas de contingencia para buscar asociaciones entre las diferentes variables. Se aplicó el estadístico Chi-cuadrado para analizar dichas asociaciones, y se aplicaron el estadístico de V de Cramer para analizar su fuerza. El valor de significación estipulado en el estudio para demostrar la asociación entre las diferentes variables fue p<0,05.

Para la puntuación global obtenida se hizo un análisis de varianza factorial (Anova): el primer factor fue la etapa educativa con dos niveles (educación primaria y educación secundaria obligatoria) y el segundo factor fue la variable formación previa (Sí-No). Se estudiaron los efectos principales utilizando "η2", y también la interacción entre variables, utilizando el estadístico de Bonferroni para conocer la significación.

## RESULTADOS

El número total de participantes fue de 240, 140 en educación primaria (EP) (5°, n= 70; 6°, n=70) y 100 en educación secundaria obligatoria (ESO) (1°, n= 50; 2°, n=50).

### Análisis descriptivo y comparativo intragrupo - EP

El grupo de educación primaria (EP) estaba formado por 79 niños (56,4%) y 61 niñas (43,6%) y la edad media fue de 10,85 ± 0,7 años. Menos de la mitad, 58 (41,4%), recibió formación en primeros auxilios antes de participar en el estudio.

Solamente seis alumnos (4,3%) consideraron tener conocimientos suficientes en primeros auxilios. Más de la mitad (77; 55%) consideraban que sus conocimientos eran bajos y el resto (55; 40,7%), ninguno. Los resultados muestran que solo 12 alumnos (8,6%) pudieron explicar de forma correcta en qué consistía el masaje cardíaco, 62 (44,3%) aseguraban conocer el DEA, 30 de ellos (21,4%) su funcionamiento y 64 (45,7%) cuál era la intención de su uso.

En cuanto a las preguntas orientadas a comprobar sus conocimientos, se observó que 10 de los alumnos (14,3%) redactaron correctamente la secuencia de actuación en el protocolo de SVB, 136 (97,1%) conocían el número de emergencias, 32 (22,9%) sabían verificar la consciencia de un accidentado y 13 (9,3%), realizar correctamente la apertura de la vía aérea (Tabla 1).

**Tabla 1:** Análisis comparativo sobre conocimientos reales en SVB por el alumnado de educación primaria (EP)

	5.º EP	6.º EP	X2	p valor
	(n=70) (%)	(n=70) (%)		
Secuencia de actuación SVB correcta	10 (14,3)	12 (17,1)	0,216	0,408
Teléfono de emergencias correcto	67 (95,7)	69 (98,6)	1,029	0,310
Verificación de conciencia correcta	14 (20,0)	18 (25,7)	0,648	0,273
Apertura de la vía aérea correcta	5 (3,5)	8 (5,7)	0,037	0,500
Evaluación de la respiración correcta	19 (27,1)	20 (28,6)	0,036	0,500
Lugar de colocación de manos correcto	59 (84,3)	51 (72,9)	2,715	0,074
Fracción de compresiones torácicas/ventilación correcta	1 (1,4)	2 (2,9)	0,314	0,500
Frecuencia de compresiones/minuto correctas	0 (0,0)	0 (0,0)	--	--
Profundidad de las compresiones torácicas correcta	11 (15,7)	21 (30,0)	4,051	0,035
Orden de ejecución del DEA correcto	11 (15,7)	12 (17,1)	0,052	0,500

Sobre la reanimación cardiopulmonar (RCP), 110 (78,6%) sabían colocar las manos correctamente en el cuerpo del accidentado, 3 (2,1%) la ratio de compresiones/ventilaciones, 0 (0,0%) el ritmo de las compresiones y 32 (22,9%) la profundidad de estas. Por último, 23 (16,4%) ordenaron correctamente la secuencia de actuación con el desfibrilador externo automático (DEA).

En este apartado también se observa que, exceptuando los conocimientos sobre la profundidad de las compresiones, donde el alumnado de 6.° responde con más acierto (11 (15,7%) vs. 21 (30,0%); p=0.03 5), en el resto de las variables analizadas no se encuentran diferencias significativas entre los dos cursos (Tabla 1).

### Análisis descriptivo y comparativo intragrupo - ESO

El grupo de educación secundaria (ESO) estaba formado por 50 niños (50,0%) y 50 niñas (50,0%) y la edad media fue de 13,0 ± 0,8 años. Más de la mitad, 54 (54,0%) recibió formación en primeros auxilios antes de participar en el estudio.

Sobre la percepción de conocimientos, 13 alumnos (13,0%) consideraron tener conocimientos suficientes en primeros auxilios. Más de la mitad (65; 65,0%) consideraban que sus conocimientos eran bajos y el resto (22; 22,0%), ninguno. Los resultados muestran que solo 9 alumnos (9,0%) pudieron explicar de forma correcta en qué consistía el masaje cardíaco, 40 (40,0%) aseguraban conocer el DEA, 30 de ellos (30,0%) su funcionamiento y 66 (66,0%) cuál era la intención de su uso.

En cuanto a las preguntas orientadas a comprobar sus conocimientos, se observó que 19 de los alumnos (19,0%) redactaron correctamente la secuencia de actuación en el protocolo de SVB, 96 (96,0%) conocían el número de emergencias, 19 (32,0%) sabían verificar la conciencia de un accidentado y 5 (5,0%), realizar correctamente la apertura de la vía aérea (Tabla 2).

**Tabla 2:** Análisis comparativo sobre conocimientos reales en SVB por el alumnado de educación secundaria (ESO)

	1ºESO	2ºESO	X2	p valor
	(n=50)	(n=50)		
Secuencia de actuación SVB correcta	7 (14,0%)	12 (24,0%)	1,624	0,154
Teléfono de emergencias correcto	48 (96,0%)	48 (96,0%)	0,000	0,691
Verificación de conciencia correcta	6 (12,0%)	13 (26,0%)	3,184	0,062
Apertura de la vía aérea correcta	1 (2,0%)	4 (8,0%)	1,895	0,181
Evaluación de la respiración correcta	13 (26,0%)	21 (42,0%)	2,852	0,069
Lugar de colocación de manos correcto	38 (76,0%)	40 (80,0%)	0,233	0,405
Fracción de compresiones torácicas/ventilación correcta	5 (10,0%)	0 (0,0%)	5,263	0,028
Frecuencia de compresiones/minuto correctas	0 (0,0%)	0 (0,0%)	--	--
Profundidad de las compresiones torácicas correcta	18 (36,0%)	12 (24,0%)	1,714	0,138
Orden de ejecución del DEA correcto	8 (16,0%)	7 (14,0%)	0,078	0,500

Sobre la reanimación cardiopulmonar (RCP), 78 (78,0%) sabían colocar las manos correctamente en el cuerpo del accidentado, 5 (5,0%) la ratio de compresiones/ventilaciones, 0 (0,0%) el ritmo de las compresiones y 30 (30,0%) la profundidad de estas. Por último, 15 (15,0%) ordenaron correctamente la secuencia de actuación con DEA.

En este apartado también se observa que, exceptuando los conocimientos sobre la ratio compresiones/ventilaciones, donde el alumnado de 1.° responde con más acierto (5 (10,0%) vs. 0 (0,0%); p=0,028), en el resto de las variables analizadas no se encuentran diferencias significativas entre los dos cursos (Tabla 2).

### Análisis comparativo intergrupo - EP vs. ESO

En el análisis estadístico realizado se muestra que existen diferencias significativas entre los dos grupos (EP vs. ESO) en tres variables estudiadas: la edad (10,85 ± 0,7 vs. 13,00 ± 0,8 años; p<0,001); la cantidad de alumnos con ningún conocimiento sobre SVB (40,7% vs. 22,0%; p=0,002) y la cantidad de alumnos con conocimientos suficientes (4,3% vs. 13,0%; p=0,014) (Tabla 3).

**Tabla 3:** Datos demográficos y percepción de conocimientos sobre SVB en ambos grupos (EP vs. ESO)

		Etapa educativa		p valor*
		EP	ESO	
		(n=140) (%)	(n=100)	
Edad (años)		10,85 (0,7)	13,00 (0,8)	<0,001
Género [n (%)]	Hombre	79 (56,4)	50 (50,0)	--
	Mujer	61 (43,6)	50 (50,0)	--
Formación previa	Sí	51 (36,4)	28 (28,1)	0,172
	Ningunos	57 (40,7)	22 (22,0)	0,002
Autopercepción de conocimientos	Algunos	77 (55,0)	65 (65,0)	0,120
	Suficientes	6 (4,3)	13 (13,0)	0,014
Conocimiento masaje cardíaco externo	Sí	12 (8,6)	9 (9,0)	0,908
Conocimiento del DEA	Sí	62 (44,3)	40 (40,0)	0,277
Funcionamiento del DEA	Sí	30 (21,4)	30 (30,0)	0,131
Intención de uso del DEA	Sí	64 (45,7)	66 (66,0)	0,069

* Prueba U de Mann Whitney

Con respecto a los conocimientos demostrados, no se observa ninguna diferencia entre grupos en las variables analizadas (Tabla 4).

**Tabla 4:** Análisis descriptivo y comparativo sobre conocimientos reales en SVB en ambos grupos (EP vs. ESO)

	EP	ESO	X2	p valor
	(n=140) (%)	(n=100) (%)		
Secuencia de actuación SVB correcta	22 (15,7)	19 (19,0)	0,445	0,310
Teléfono de emergencias correcto	136 (97,1)	96 (96,0)	0,236	0,444
Verificación de consciencia correcta	32 (22,9)	19 (19,0)	0,107	0,437
Apertura de la vía aérea correcta	13 (9,3)	5 (5,0)	1,544	0,160
Evaluación de la respiración correcta	39 (27,9)	34 (34,0)	1,040	0,190
Lugar de colocación de manos correcto	110 (78,6)	78 (78,0)	0,011	0,519
Ratio de compresiones torácicas/ventilación correcta	3 (2,1)	5 (5,0)	1,478	0,197
Frecuencia de compresiones/minuto correctas	0 (0,0)	0 (0,0)	--	--
Profundidad de las compresiones torácicas correcta	32 (22,9)	30 (30,0)	1,553	0,137
Orden de ejecución del DEA correcto	23 (16,4)	15 (15,0)	0,089	0,455

Respecto a la puntuación media global obtenida (Tabla 5), los resultados muestran que existe un efecto principal en el factor formación previa [F(1,515) = 15,804; gl=1; p=0,001, η2=0,062], siendo más elevada la puntuación global en el alumnado que había recibido formación en materia de primeros auxilios antes de participar en el estudio.

**Tabla 5:** Estadísticos descriptivos de la puntuación global de aciertos en función de la etapa educativa y la formación previa

		Total (n=240)		Educación primaria (n=140)		Educación secundaria (n=100)	
	Formación previa	M	DE	M	DE	M	DE
Puntuación global	Sí (n=161)	3,41	1,35	3,24	1,40	3,71	1,21
No (n=79)	2,73	1,10	2,74	1,10	2,72	1,24

Nota: M = media; DE = desviación estándar

Dentro de los dos grupos también se observa un efecto principal en el factor formación previa, en EP [F(1,494) = 5,288; gl=1; p=0,023, 112=0,037], y en ESO [F(1,532) = 12,949; gl=1; p=0,001, η2=0,117]. Sin embargo, no se han encontrado diferencias estadísticamente significativas para el factor etapa educativa (p=0,637).

## DISCUSIÓN

Tras la realización de este estudio se ha comprobado, en primer término, que el alumnado de educación primaria apenas tiene conocimientos sobre soporte vital básico o primeros auxilios. Exceptuando el conocimiento del número de emergencias, prácticamente todos los alumnos evaluados tienen conocimientos muy pobres. Esto guarda relación con lo obtenido en estudios previos, como el de Navarro-Patón et al. 18, donde el alumnado de educación primaria también obtuvo valores bajos en conocimientos sobre SVB.

Sin embargo, lo más destacable es lo ocurrido en el alumnado de educación secundaria obligatoria, en el que se ha observado que los resultados son similares a los de la anterior etapa educativa. Esto sorprende ya que el currículo estatal 15 y todos los currículos autonómicos incluyen contenidos relacionados con los primeros auxilios más amplios que en educación primaria, incluyendo la conducta PAS, el protocolo de SVB, la realización de la RCP y el uso del DEA 16. Además, en estudios como el de Navarro-Patón et al. 18 se observó que el alumnado de educación secundaria reportaba mayor conocimiento que el de educación primaria.

Por último, debemos destacar que el alumnado con formación previa, ya sea porque la ha recibido en el colegio o fuera de este, obtiene mejor resultados en las preguntas relacionadas con la valoración de la conciencia o la respiración, lo que refleja que esta formación sí es necesaria y efectiva para que todas las personas, desde la infancia, sean capaces de ayudar ante un accidente 5.

En este estudio, la formación recibida, además de escasa, fue similar entre el grupo de educación primaria y el de educación secundaria, lo que se contrapone a lo encontrado en estudios anteriores, en los cuales la formación de los alumnos más mayores suele ser también mayor 18.

Esto puede ser debido a que los datos se recogieron en centros diferentes y que los profesores de dichos centros no tengan los mismos recursos o conocimientos para impartir dichos contenidos 19. Esta falta de formación en el profesorado también pudo suponer el déficit de conocimientos del alumnado.

Debido a los valores tan bajos de conocimientos del alumnado de este y otros estudios, se hace imperiosa la necesidad de mejorar su formación. Se ha demostrado que los niños pueden aplicar medidas de primeros auxilios, reconocer situaciones de emergencia y realizar una llamada de auxilio ante una emergencia 20.

Estudios previos han demostrado que sesiones cortas de formación repetidas una o dos veces en el curso académico podrían mejorar y mantener de forma significativa los conocimientos teóricos y prácticos en materia de primeros auxilios y RCP 21-23.

Además, es imprescindible que el profesorado aumente sus competencias en materia de primeros auxilios por dos motivos. En primer lugar, porque se ha demostrado que es el colectivo idóneo para formar al alumnado si se capacitan previamente 22,24,25 y, en segundo lugar, porque la Ley Orgánica 3/2020, de 29 de diciembre, que modifica la Ley Orgánica 2/2006, de 3 de mayo, de Educación (LOMLOE) y su concretización a nivel estatal mantienen el tratamiento de estos contenidos de forma obligatoria (en la asignatura de Educación Física), en el tercer ciclo de educación primaria y toda la etapa de educación secundaria obligatoria (Tabla 6).

**Tabla 6:** Contenidos de primeros auxilios impartidos a nivel estatal (RD157/2022; RD 217/2022)

Curso	Competencia específica	Criterios de evaluación	Contenidos
5º EP 6º EP	CE1. Adoptar un estilo de vida activo y saludable, seleccionando e incorporando intencionalmente actividades físicas y deportivas en las rutinas diarias…	CE 1.3 Adoptar medidas de seguridad antes, durante y después de la práctica de actividad física, reconociendo los contextos de riesgo y actuando con precaución ante ellos.	B. Prevención de accidentes y lesiones en las prácticas motrices: calentamiento general y vuelta a la calma, dosificación del esfuerzo y recuperación. Importancia de respetar las normas de seguridad. B. Actuaciones básicas ante accidentes y lesiones durante la práctica de actividades físicas. Posición lateral de seguridad. Conductas PAS (proteger, avisar, socorrer).
1º ESO 2º ESO	CE1. Adoptar un estilo de vida activo y saludable, seleccionando e incorporando intencionalmente actividades físicas y deportivas en las rutinas diarias…	CE 1.5 Adoptar de manera responsable y con progresiva autonomía medidas generales para la prevención de lesiones antes, durante y después de la práctica de actividad física, aprendiendo a reconocer situaciones de riesgo para actuar preventivamente. CE 1.6 Actuar de acuerdo con los protocolos de intervención ante accidentes derivados de la práctica de actividad física, aplicando medidas básicas de primeros auxilios.	B. “Prevención de accidentes en las prácticas motrices: calzado deportivo y ergonomía. Medidas de seguridad en actividades físicas dentro y fuera del centro escolar”. B. “Actuaciones ante accidentes durante la práctica de actividades físicas. Conducta PAS (proteger, avisar, socorrer). Protocolo 112. Soporte vital básico (SVB)”.
3º ESO 4º ESO	CE1. Adoptar un estilo de vida activo y saludable, seleccionando e incorporando intencionalmente actividades físicas y deportivas en las rutinas diarias…	CE 1.3 Adoptar de manera responsable y autónoma medidas específicas para la prevención de lesiones antes, durante y después de la práctica de actividad física, aprendiendo a reconocer situaciones de riesgo para actuar preventivamente. CE 1.4 Actuar de acuerdo con los protocolos de intervención ante situaciones de emergencia o accidentes, aplicando medidas específicas de primeros auxilios.	B. “Prevención de accidentes en las prácticas motrices. Gestión del riesgo propio y del riesgo de los demás. Medidas colectivas de seguridad”. B. “Actuaciones ante accidentes. Reanimación mediante desfibrilador automático (DEA) o semiautomático (DESA). Protocolo RCP (reanimación cardiopulmonar). Técnicas específicas e indicios de accidentes cardiovasculares (maniobra de Heimlich, señales de ictus y similares)”.

En definitiva, los alumnos participantes del estudio, tanto de educación primaria como de educación secundaria, no poseen los conocimientos necesarios para poder actuar y solventar una situación de emergencia, y en el caso de que los tengan, no presentan las condiciones necesarias para poder realizar una maniobra de manera eficaz, sin poner la vida en peligro de nadie.

De esta manera, es importante como futuros maestros tener en cuenta los resultados de este estudio, ya que podemos dar solución a la hipótesis principal de esta investigación, y no solo tener en cuenta los conocimientos que poseen los alumnos, sino plantearse que cierta problemática recae en el profesorado que debería impartir dichos contenidos. Por lo que, antes de eso deberíamos preocuparnos por la adquisición eficaz y segura por parte de los futuros maestros en materia de PPAA.

La educación en primeros auxilios y RCP debe ser una prioridad en la formación integral de los estudiantes, preparando a futuras generaciones para ser agentes activos en la protección y cuidado de la salud pública ♥
